# Regional variations in incidence and treatment trends of Achilles tendon ruptures in Finland: a nationwide study

**DOI:** 10.2340/17453674.2024.41089

**Published:** 2024-07-17

**Authors:** Marjukka HALLINEN, Henri SALLINEN, Heli KESKINEN, Markus MATILAINEN, Elina EKMAN

**Affiliations:** 1 Department of Orthopedics and Traumatology, Turku University Hospital, Turku; 2 Department of Orthopedics and Traumatology, Satasairaala Central Hospital; 3 University of Turku, Turku, Finland

## Abstract

**Background and purpose:**

The aim of our study was to assess the regional variations in Achilles tendon rupture incidence and treatment methods in Finland during the period 1997–2019.

**Methods:**

The Finnish National Hospital Discharge Register (NHDR) and the Finnish Register of Primary Health Care Visits (PHCR) were searched to identify all adult patients diagnosed with Achilles tendon rupture during our study period. The population-based annual incidence and incidences of surgically and non-surgically treated Achilles tendon ruptures were calculated for each hospital district.

**Results:**

Achilles tendon rupture incidence increased from 17.3 per 10^5^ person-years in 1997 to 32.3 per 10^5^ in 2019. The mean incidence of Achilles tendon ruptures ranged from 26.4 per 10^5^ (North Savo) to 37.2 per 10^5^ (Central Ostrobothnia). The incidence of Achilles tendon ruptures increased in all areas. The proportion of non-surgical treatment of Achilles tendon ruptures ranged in 1997 from 7% (Vaasa) to 67% (Åland) and in 2019 from 73% (Southwest Finland) to 100% (East Savo, Kainuu, Länsi-Pohja, Åland). During the study period, a shift towards non-surgical treatment was evident in all hospital districts.

**Conclusion:**

Regional variations in Achilles tendon rupture incidence exist in Finland; however, the incidence increased in all areas during the follow-up period. More Achilles tendon rupture patients are currently being treated non-surgically throughout the country.

During recent decades, the incidence of Achilles tendon rupture has increased globally from 6.7–27.0 to 10.8–32.3 per 105 between the 1990s and the first decades of the 21st century [1-4]. The average age at Achilles tendon rupture has increased, from 37.0 before 1970 to 42.1 in 2014 [5]. Furthermore, although male sex has been reported as a risk factor for Achilles tendon rupture, the proportion of females suffering from Achilles tendon ruptures also increased from 1953 to 2014 [5]. Notably, Achilles tendon ruptures occur mostly while participating in high-impact sports, typically badminton, basketball, or football [6,7].

Previously most Achilles tendon ruptures have been treated surgically, but recent studies have found that non-surgical treatments deliver comparable functional outcomes without the risk of surgical complications such as infections or sural nerve injuries [8-11]. This has led to an increase in non-surgical treatment of Achilles tendon ruptures [2-4,12].

The healthcare system in Finland is publicly funded and indications to treat orthopedic trauma should be uniform. Therefore, regional variations in treatment methods should be minimal. Regarding Achilles tendon ruptures, only a limited number of studies concerning their regional variations in incidence and treatment methods have been conducted [3,12,13]. To address this gap in the literature, the aim of our study was to assess the regional variations in Achilles tendon rupture incidence and treatment methods in Finland between 1997 and 2019 based on data from 2 national registers: the National Hospital Discharge Register (NHDR) and the Finnish Register of Primary Health Care Visits (PHCR).

## Methods

This register study is based on data obtained from the National Hospital Discharge Register (NHDR) and the Finnish Register of Primary Health Care Visits (PHCR), both of which are maintained by the National Institute for Health and Welfare. Data reporting to the NHDR, which was founded in 1967, is obligatory for all public and private hospitals in Finland. Similarly, the PHCR, which has been operational since 2011, receives data on all patient encounters within the public primary healthcare system (primary health care centers) in Finland. These registers contain data on the patient’s age, sex, domicile, external cause of injury, type of injury, primary and secondary diagnoses, type of hospital (public or private), duration of hospital stay, and possible operations performed during the hospital stay. Notably, the NHDR has exhibited substantial validity, especially in the case of orthopedic trauma [14-16]. Validation has not been studied for Achilles tendon ruptures. For the NHDR the degree of completeness is over 95% [14] and accuracy 88–80% [15,16].

**Figure 1:**
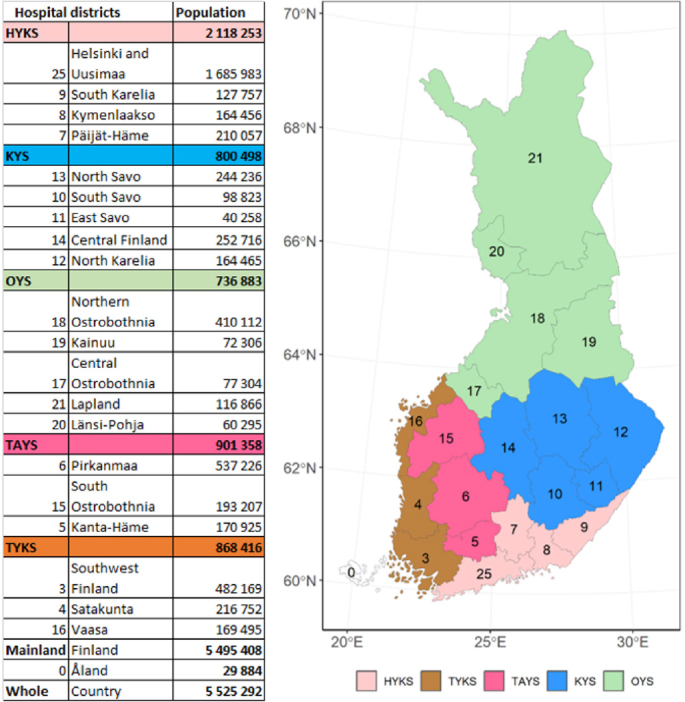
Map of specific catchment areas of healthcare and hospital districts in Finland. Population on December 31, 2019. University hospital districts in Finland: Helsinki University Hospital (HYKS), Turku University Hospital (TYKS), Tampere University Hospital (TAYS), Kuopio University Hospital (KYS) and Oulu University Hospital (OYS).

Finland is divided into 21 hospital districts (Figure 1). The role of these districts is to provide special healthcare services, such as surgical treatment, to municipalities in the region. The hospital districts are gathered into 5 University Hospital districts. We used the division into hospital districts to assess possible regional differences in the incidence and treatment methods of Achilles tendon ruptures.

Patients with Achilles tendon ruptures were identified by searching both databases using the primary or secondary diagnosis code of S86.0—injury of Achilles tendon (International Classification of Diseases, 10th Revision [ICD-10]). To identify all surgically and non-surgically treated patients, the NHRD was searched for all records with the procedural code NHL10, which refers to “suture or reinsertion of Achilles tendon” (Nordic Classification of Surgical Procedures [NCSP]). Following this identification process, all duplicates between the 2 registers were excluded. Notably, the records of diagnosis code S86.0 were included in the surgical incidence calculation only if the operation was performed within 30 days of the injury. A more detailed description has been published in a previous study [2]. This criterion was considered to exclude other operations performed for treating Achilles tendon injuries, such as salvage of non-surgical treatment failures or reoperation. Moreover, all pediatric patients (younger than 16 years of age) were excluded from the study. Since the NHDR and PHCR do not report injury laterality, only the first recorded injury was included. Specifically, data on patient age, sex, procedures performed during the hospital stay, and hospital district of the patient were collected. Subsequently, incidences were calculated based on the annual adult population size (persons aged ≥ 16 years) of the hospital district in question, as obtained from Statistics Finland (www.stat.fi) [17]. Patients without information regarding the hospital district were excluded (29 patients).

### Statistics

Because the incidences were based on the entire population rather than cohort-based estimates, confidence intervals were not reported. Incidence calculations and other statistical reporting were performed using R software version 4.3.0 (R Core Team; R Foundation for Statistical Computing, Vienna, Austria).

### Ethics, data sharing, funding, and disclosures

Ethical approval for the study was granted by the Finnish National Institute of Health and Wellness (study permit number THL/2266/5.05.00/2019). The authors declare no conflicts of interest. We received research funding from the Finnish government to finance the research. The data that support the findings of this study is available from the corresponding author upon reasonable request. Complete disclosure of interest forms according to ICMJE are available on the article page, doi: 10.2340/17453674.2024.41089

## Results

**Figure 2:**
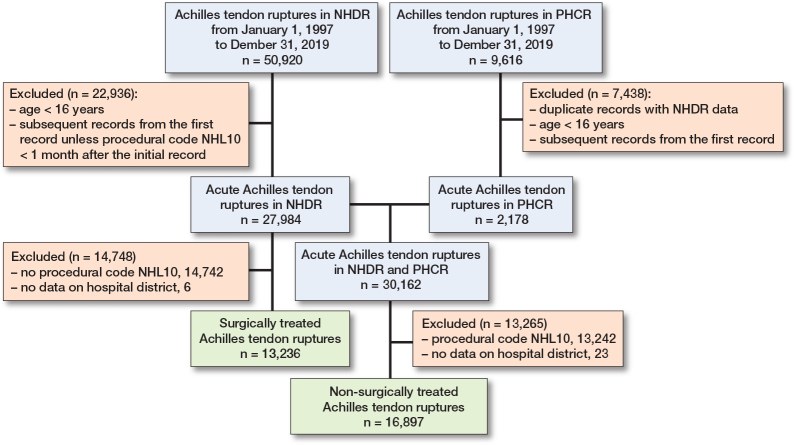
Patient inclusion flowchart.

**Figure 3:**
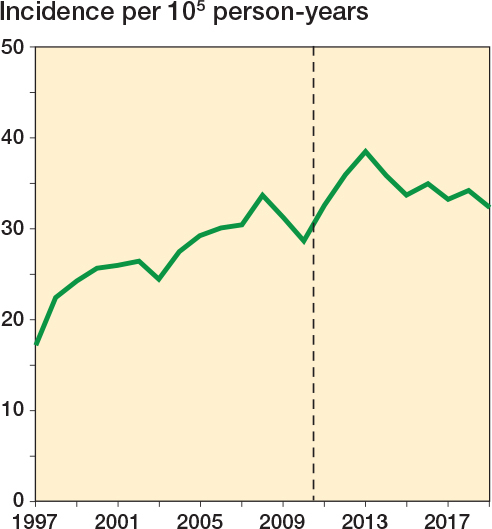
Incidence of Achilles tendon rupture between 1997 and 2019; the vertical line indicates the introduction of the PHCR and its data.

**Figure 4:**
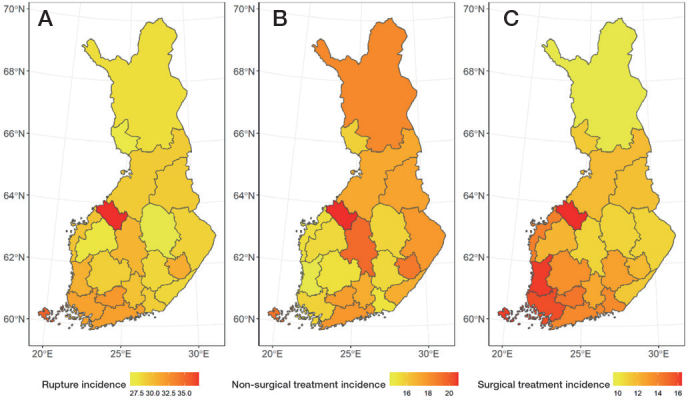
Map of Finland, divided into 21 hospital districts, presenting: (A) total incidence of Achilles tendon rupture per 10^5^; (B) non-surgical treatment incidence per 10^5^; and (C) surgical treatment incidence per 10^5^ between 1997 and 2019.

**Figure 5:**
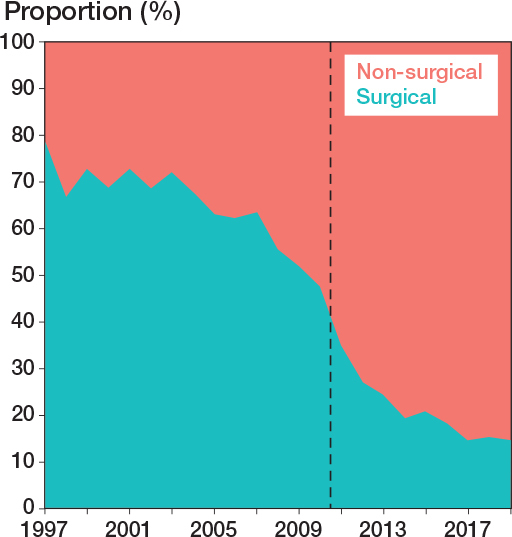
Proportions of surgical and non-surgical treatment of Achilles tendon rupture between 1997 and 2019; vertical line indicates the introduction of the PHCR and its data.

30,133 Achilles tendon ruptures were identified during the 23-year study period (Figure 2). The total Achilles tendon rupture incidence was 30.1 per 10^5^ person-years. Overall, the incidence of Achilles tendon rupture increased from 17.3 per 10^5^ in 1997 to 32.3 per 10^5^ in 2019 (Figure 3). Furthermore, differences could be observed in the mean incidence of Achilles tendon ruptures among the different regions of Finland (Figure 4A), which ranged from 26.4 per 10^5^ (North Savo) to 37.2 per 10^5^ (Central Ostrobothnia). The incidence of Achilles tendon rupture increased most in East Savo (from 4.8 in 1997 to 36.7 per 10^5^ in 2019) and least in Northern Ostrobothnia (from 18.5 in 1997 to 28.3 per 10^5^ in 2019). The mean incidence of Achilles tendon rupture grew in every hospital district during the study period (see Supplementary data). A change in favor of non-surgical treatment was evident, especially between the years 2008 and 2019, as the percentage of surgical treatment decreased from 55% to 15% and that of non-surgical treatment increased correspondingly from 45% to 85% (Figure 5). Furthermore, the mean age of patients with Achilles tendon rupture increased during the 23-year period (44 years in 1997 vs 51 years in 2019). During the entire study period, surgically treated patients were largely younger than those treated non-surgically (44 vs 53 years). This was true in all regions (surgically treated group 42–49 years, non-surgically treated group 49–56 years). The mean age of all patients in this study was 49 years. Patients were youngest in the Helsinki and Uusimaa region (48 years) and oldest in East Savo (53 years). Patients with Achilles tendon ruptures were more often male (75%) than female (25%) in all regions. The difference was also clear in the surgically treated group (male 80% vs female 20%). No significant differences in the sex proportion between the hospital districts were found in any of the groups.

**Figure 6:**
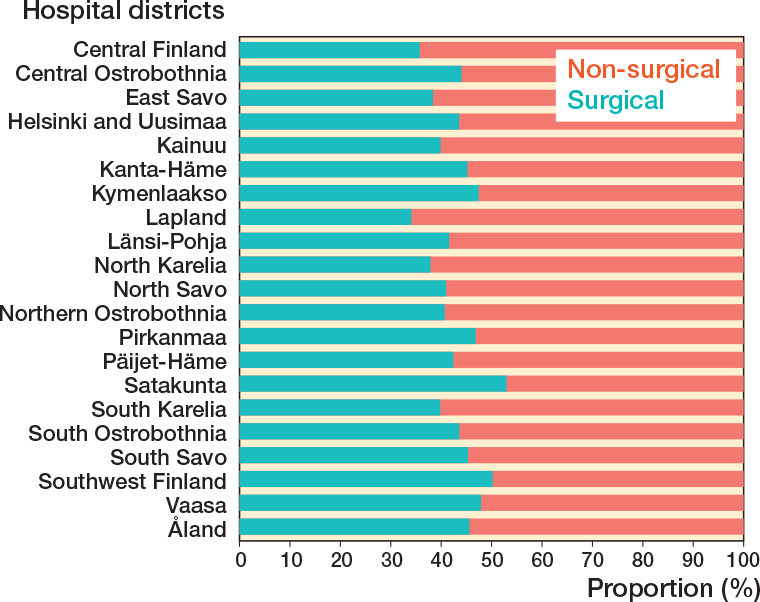
Proportions of non-surgical and surgical treatment in each of the 21 hospital districts between 1997 and 2019.

Furthermore, regional differences between the incidence of surgical and non-surgical treatments could be identified (Figures 4B, 4C, and 6). The proportion of non-surgical treatment of Achilles tendon ruptures ranged in 1997 from 7% (Vaasa) to 67% (Åland) and in 2019 from 73% (Southwest Finland) to 100% (East Savo, Kainuu, Länsi-Pohja, Åland). When pooling together the data from the whole 23-year study period, the proportion of non-surgical treatment of Achilles tendon ruptures was lowest in Satakunta (47%) and highest in Lapland (66%). The proportion of surgical treatment ranged in 1997 from 33% (Åland) to 93% (Vaasa) and in 2019 from 0% (East Savo, Kainuu, Länsi-Pohja, Åland) to 27% (Southwest Finland). The biggest change towards non-surgical treatment was in Kainuu (from 8% to 100%) and smallest in Åland (from 67% to 100%). The proportion of non-surgical treatment grew in all hospital districts during the study period (see Supplementary data). When pooling together the data from the whole 23-year study period, non-surgical treatment was generally preferred across the country, while surgical treatment was preferred only in Satakunta (53%) and Southwest Finland (50%).

**Figure 7:**
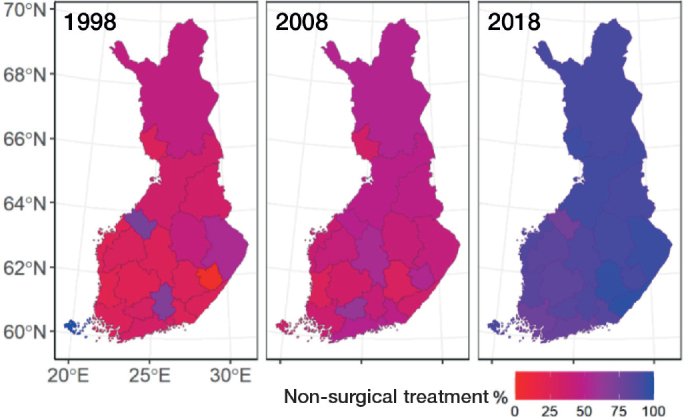
Map of Finland, divided into the 21 hospital districts, presenting the proportions of non-surgical treatment across the hospital districts in 1998, 2008, and 2018.

**Figure 8:**
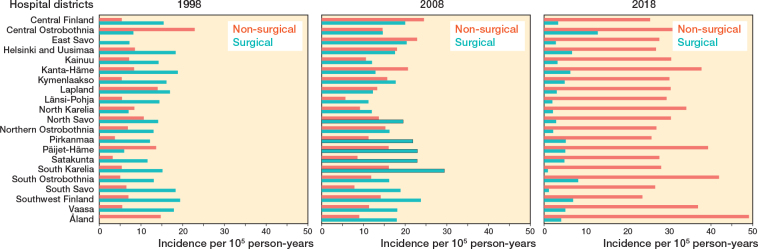
Incidence of surgical treatment and non-surgical treatment across the hospital districts in 1997, 2008, and 2018.

On a larger scale, surgical treatment was more common during the study period in the hospital districts of southern and western Finland compared with those of northern and eastern Finland (46% vs 40%). The patients were younger in southern and western Finland (mean age 48.9 years (standard deviation [SD] 15.3) vs 49.4 years [SD 15.4]). The total incidence of Achilles tendon ruptures (30.3 and 29.4 per 10^5^) and the incidence of non-surgical treatment (17.7 and 16.4) in these 2 regions were similar. Although there were differences in the proportions of non-surgical and surgical treatment among the hospital districts during the study period, a shift towards non-surgical treatment was evident across all hospital districts on observing the data in terms of 10-year intervals (Figures 7 and 8).

## Discussion

The aim of our study was to assess the regional variations in Achilles tendon rupture incidence and treatment methods in Finland during the period 1997–2019. Distinct regional differences were observed in both the incidence and the treatment methods of Achilles tendon rupture in Finland. The mean incidence of Achilles tendon rupture was highest in Central Ostrobothnia and lowest in North Savo, and the incidence increased in all regions during the follow-up period. Meanwhile, surgical treatment was preferred over non-surgical treatment in Southwest Finland and Satakunta. Moreover, differences between the treatment methods during the study period were observed to have shifted toward non-surgical treatment nationwide. Although there are no written national recommendations, the research evidence for non-surgical treatment of Achilles tendon rupture is strong and should guide treatment throughout the country. The decrease of surgical treatment in Finland coincides with the increase of research data regarding Achilles tendon rupture treatment.

Earlier studies have also reported the existence of regional differences in the mean incidence of Achilles tendon ruptures, especially when comparing rural and urban regions. In Ontario, Canada, a higher probability of Achilles tendon rupture was reported in urban neighborhoods [12]. Similarly, a higher incidence of Achilles tendon ruptures in urban regions has also been reported in Finland [13]. The present study confirms these findings, with the incidence of Achilles tendon ruptures found to be higher in Southern Finland, an area with higher population density. However, some conflict still exists in this context: Denmark exhibited no difference between its rural and urban areas [3]. Notably, Finland is a sparsely populated country marked by uneven population distribution; a majority of the population is concentrated in the small and urban southwestern coastal plain. High population density areas generally contain more sports facilities, indicating more possibilities to participate in activities that are known to carry a risk of Achilles tendon rupture, such as badminton, football, and basketball [6]. In contrast, in Denmark the risk activities are performed both in rural and urban areas.

During the study period, surgical treatment was found to be more common in hospital districts in southern and western Finland compared with those in northern and eastern Finland. In these areas, patients were slightly younger (48.9 years [SD 15.3] vs 49.4 years [SD 15.4]), which may be a contributing factor to the higher prevalence of surgical treatment. Notably, previous studies on Achilles tendon rupture incidence have not compared the incidence of treatment methods among regions [3,12,13].

Treatment accessibility can significantly guide patients’ choice. As a shared decision is often considered in the case of Achilles tendon ruptures, a patient may choose the easier treatment method, i.e., non-surgical treatment that can be carried out in healthcare centers. This is especially tempting in Lapland, where the distance to the nearest hospital is often significant. Furthermore, according to the literature, different attitudes and beliefs regarding indications for surgery are the most important reasons for regional variations in the implementation of surgical procedures [18]. Discretionary and preference-sensitive procedures tend to vary considerably more than procedures for which clinical decisions are constrained to a narrow range of options. For example, there is no clear consensus in the treatment of proximal humerus fractures, which exhibit higher regional variations in the choice of treatment methods [19]. In contrast, in the case of hip fractures, there is a clear consensus that they need to be operated on to avoid mortality, as a result of which there is practically no variation in its treatment methods [20]. In this sense, Achilles tendon rupture treatment may be classified as a discretionary procedure, although growing evidence from randomized controlled studies should contribute to forming a national guideline on the treatment of Achilles tendon ruptures, thus diminishing the effect of attitudes and beliefs and, in turn, minimizing regional differences. In this context, our study shows that the treatment methods for Achilles tendon ruptures shifted towards non-surgical treatment across the country during the study period.

The mean incidence of Achilles tendon ruptures has been increasing globally [3,4]. Naturally, this phenomenon has also been confirmed in Finland [2]. We found that, along with the increasing incidence, the mean age of patients with Achilles tendon rupture has increased as well (from 44 in 1997 to 51 years in 2019 years). This points to a clear transition towards a much older population structure in Finland, as is the case in other Western countries. This demographic phenomenon is characterized by a decrease in birth rate, a decrease in mortality rate, and a higher life expectancy in the population [21]. Therefore, one possible explanation for the increasing incidence of Achilles tendon ruptures could be the growing number of older adults participating in high-demand sports [22,23].

### Strengths

This is a population-based study of Finland and its presentation of variations in the incidence and treatment methods of Achilles tendon ruptures among hospital districts in the country.

### Limitations

As the NHDR and PHCR do not report injury laterality, only the first recorded injury was included in our study, thus excluding possible acute Achilles tendon rupture of the other limb, which may have led to a slight underestimation of the injury incidence. However, we do not believe that this creates a significant bias, as this exclusion was implemented on the entire data, including those of all regions, and therefore it does not affect regional treatment trends. Furthermore, PHCR data collection began in 2011. As primary healthcare does not offer surgical treatment, the introduction of the PHCR data resulted in a rise in the incidence of non-surgically treated Achilles tendon rupture, accounting for a patient population that was previously not registered elsewhere. During the study period, 16,897 non-surgically treated acute Achilles tendon ruptures were registered in the NHDR and PHCR, among which 2,178 cases were treated non-surgically in primary healthcare centers and were thus registered only in the PHCR [2]. Although this number may be considered relatively small, the introduction of PHCR does explain some of the increase in non-surgical treatment, but not all. Moreover, an increasing incidence of non-surgical treatment was observed in both registers during the study period. The NHDR has exhibited substantial validity in orthopedic traumas, but the degree of completeness of the PHCR has not been studied. Validity has not been separately defined for Achilles tendon rupture, but we believe it is similar to other orthopedic traumas. The registers are also at risk of incorrect entries by miscoding; however, the Achilles tendon rupture diagnosis is quite unambiguous to a physician and therefore we do not think this will cause a significant distortion.

### Conclusion

This study revealed the existence of regional variations in the incidence of Achilles tendon rupture in Finland. Furthermore, it highlighted that the total incidence of Achilles tendon rupture has increased, from 17.3 per 10^5^ in 1997 to 32.3 per 10^5^ in 2019, along with an increase in the mean age of patients. The incidence of Achilles tendon rupture increased most in East Savo (change of 31.9 per 10^5^) and least in Northern Ostrobothnia (change of 9.9 per 10^5^). Additionally, although regional differences were identified in the preferred treatment method for Achilles tendon ruptures in Finland, this trend has encountered a turning point towards non-surgical treatment in recent years, with the corresponding changes visible in all hospital districts in Finland.

### Supplementary data

Tables 1–4 are available as Supplementary data on the article home page: 10.2340/17453674.2024.41089

## Supplementary Material



## Notes

All authors have accepted responsibility for the entire content of this manuscript and consented to its submission to the journal, reviewed all the results and approved the final version of the manuscript. EE: conceived the idea and supervised the project. MM: verified the analytic methods. MH and HS: wrote the manuscript in consultation with EE and HK. All authors discussed the results and contributed to the final manuscript.

Handling co-editors: Li Felländer-Tsai and Philippe Wagner

*Acta* thanks anonymous reviewers for help with peer review of this manuscript.
