# Supplementary material for: Regional variations in incidence and treatment trends of Achilles tendon ruptures in Finland: a nationwide study
Source: Acta Orthop. 2024 Jul 17;95:401–6. doi: 10.2340/17453674.2024.41089 (PMC11253709; doi:10.2340/17453674.2024.41089)
Supplement: Supplementary file 1 [file ActaO-95-41089-s1.pdf]

## Supplementary Table 1. Non-surgical treatment incidence per $10^5$ person-years

| year | Central Finland | Central Ostrobothnia | East Savo | Helsinki and Uusimaa | Kainuu | Kanta-Häme | Kymenlaakso | Lapland | Länsi-Pohja | North Karelia | North Savo | Northern Ostrobothnia | Pirkanmaa | Päijät-Häme | Satakunta | South Karelia | South Ostrobothnia | South Savo | Southwest Finland | Vaasa | Åland |
|---|---|---|---|---|---|---|---|---|---|---|---|---|---|---|---|---|---|---|---|---|---|
| 1997 | 3.2 | 6.5 | 2.4 | 4.3 | 1.4 | 2.3 | 3.3 | 8.9 | 3.6 | 8.3 | 1.4 | 3.6 | 2.4 | 5.3 | 1.6 | 2.7 | 3.1 | 5.3 | 2.8 | 1.6 | 9.9 |
| 1998 | 5.3 | 22.8 | 0.0 | 8.5 | 7.1 | 8.3 | 5.4 | 13.9 | 5.4 | 8.4 | 10.6 | 6.8 | 3.7 | 13.6 | 3.1 | 5.3 | 5.0 | 6.4 | 6.9 | 5.4 | 14.7 |
| 1999 | 5.3 | 16.3 | 0.0 | 8.6 | 10.0 | 7.5 | 4.7 | 9.0 | 3.6 | 4.2 | 7.3 | 1.8 | 6.5 | 7.1 | 4.7 | 6.2 | 3.7 | 1.1 | 7.7 | 5.4 | 19.4 |
| 2000 | 10.0 | 13.1 | 9.7 | 9.9 | 1.4 | 7.5 | 5.4 | 2.0 | 7.3 | 4.2 | 6.8 | 12.0 | 7.3 | 8.8 | 5.2 | 8.9 | 5.0 | 5.4 | 8.7 | 4.6 | 0.0 |
| 2001 | 6.8 | 13.1 | 9.8 | 8.0 | 11.6 | 6.7 | 10.1 | 11.2 | 3.7 | 5.6 | 7.3 | 5.9 | 6.9 | 8.2 | 6.3 | 4.4 | 0.6 | 6.5 | 6.5 | 3.1 | 9.6 |
| 2002 | 15.0 | 13.1 | 14.7 | 9.8 | 13.1 | 8.2 | 3.4 | 7.2 | 7.4 | 9.8 | 8.8 | 8.7 | 9.2 | 9.4 | 3.7 | 4.4 | 5.6 | 1.1 | 5.4 | 5.4 | 4.8 |
| 2003 | 6.7 | 8.2 | 4.9 | 8.0 | 11.7 | 6.7 | 6.8 | 5.1 | 12.9 | 9.8 | 4.9 | 7.9 | 4.6 | 5.8 | 5.3 | 8.0 | 2.5 | 2.2 | 7.2 | 7.7 | 4.7 |
| 2004 | 11.8 | 14.7 | 14.8 | 9.3 | 7.4 | 8.8 | 11.5 | 9.2 | 5.5 | 10.5 | 9.2 | 5.5 | 7.5 | 7.6 | 11.6 | 6.2 | 6.2 | 6.6 | 8.5 | 7.7 | 9.4 |
| 2005 | 14.2 | 3.3 | 14.9 | 12.5 | 16.3 | 13.9 | 5.4 | 5.1 | 1.8 | 14.7 | 10.7 | 7.8 | 9.5 | 12.2 | 12.7 | 15.1 | 8.1 | 8.8 | 10.1 | 6.9 | 4.6 |
| 2006 | 11.6 | 14.6 | 5.0 | 14.6 | 23.8 | 12.3 | 11.5 | 13.3 | 3.7 | 11.9 | 8.8 | 9.1 | 9.6 | 8.7 | 12.2 | 8.0 | 8.7 | 3.3 | 6.9 | 18.3 | 4.6 |
| 2007 | 14.1 | 16.2 | 15.1 | 13.5 | 12.0 | 16.5 | 8.1 | 11.2 | 5.5 | 11.2 | 4.9 | 12.0 | 7.8 | 6.9 | 9.5 | 14.2 | 9.9 | 11.0 | 9.2 | 11.4 | 13.6 |
| 2008 | 24.4 | 14.5 | 22.8 | 18.0 | 10.5 | 20.6 | 15.6 | 13.2 | 5.6 | 9.1 | 13.6 | 15.2 | 11.1 | 16.0 | 8.5 | 16.0 | 11.8 | 7.7 | 14.1 | 11.3 | 9.0 |
| 2009 | 16.8 | 16.1 | 15.3 | 16.8 | 13.5 | 17.6 | 12.2 | 12.2 | 7.4 | 4.9 | 12.1 | 19.3 | 14.1 | 15.3 | 11.7 | 10.7 | 17.3 | 12.2 | 17.6 | 8.9 | 17.7 |
| 2010 | 16.2 | 20.9 | 28.2 | 15.6 | 16.6 | 16.8 | 12.9 | 16.2 | 11.1 | 11.9 | 8.2 | 20.5 | 13.1 | 17.5 | 12.2 | 13.3 | 8.0 | 11.1 | 18.0 | 13.3 | 13.1 |
| 2011 | 22.5 | 11.2 | 28.3 | 22.8 | 21.3 | 18.8 | 9.5 | 20.2 | 16.8 | 28.1 | 24.7 | 21.9 | 19.4 | 21.4 | 23.4 | 23.1 | 20.4 | 15.6 | 22.3 | 13.2 | 17.2 |
| 2012 | 17.1 | 17.6 | 18.1 | 28.8 | 30.6 | 25.6 | 19.7 | 46.4 | 43.0 | 33.7 | 26.1 | 34.2 | 21.6 | 20.2 | 17.5 | 41.0 | 25.9 | 30.2 | 18.1 | 23.4 | 29.9 |
| 2013 | 32.0 | 38.3 | 31.3 | 30.4 | 24.6 | 22.8 | 23.1 | 31.2 | 33.9 | 36.5 | 29.9 | 31.1 | 24.9 | 21.8 | 31.4 | 36.5 | 32.1 | 24.7 | 25.4 | 32.0 | 33.9 |
| 2014 | 32.5 | 44.7 | 28.9 | 27.1 | 27.9 | 24.9 | 29.3 | 37.3 | 28.5 | 37.2 | 28.4 | 28.4 | 29.4 | 26.3 | 28.2 | 25.0 | 29.0 | 31.5 | 31.0 | 21.7 | 54.7 |
| 2015 | 34.3 | 23.9 | 34.5 | 25.5 | 25.0 | 32.5 | 33.6 | 38.3 | 30.6 | 24.6 | 24.0 | 26.4 | 27.4 | 28.6 | 23.0 | 24.2 | 26.0 | 33.9 | 23.9 | 21.7 | 12.5 |
| 2016 | 45.8 | 22.3 | 48.2 | 27.2 | 25.2 | 31.9 | 36.5 | 25.2 | 25.0 | 26.1 | 23.6 | 30.0 | 28.4 | 32.5 | 21.4 | 20.7 | 22.4 | 31.8 | 30.0 | 26.0 | 33.3 |
| 2017 | 34.1 | 41.6 | 37.9 | 26.7 | 31.8 | 22.3 | 21.5 | 27.3 | 38.8 | 32.5 | 26.0 | 26.1 | 27.0 | 28.6 | 29.6 | 35.2 | 29.9 | 40.1 | 27.9 | 33.2 | 28.9 |
| 2018 | 25.4 | 40.1 | 27.6 | 26.8 | 30.5 | 37.7 | 30.1 | 30.3 | 29.4 | 34.1 | 30.4 | 26.9 | 25.7 | 39.3 | 27.6 | 28.1 | 41.9 | 26.6 | 23.5 | 36.9 | 49.1 |
| 2019 | 35.4 | 45.1 | 36.7 | 25.1 | 35.6 | 28.7 | 31.8 | 29.4 | 37.6 | 32.1 | 31.0 | 24.0 | 24.0 | 26.5 | 22.9 | 35.5 | 32.7 | 38.6 | 22.9 | 27.4 | 32.6 |
| Change 2019-1997 | 32.2 | 38.6 | 34.3 | 20.8 | 34.2 | 26.4 | 28.5 | 20.4 | 34.0 | 23.7 | 29.6 | 20.4 | 21.6 | 21.1 | 21.3 | 32.9 | 29.6 | 33.3 | 20.2 | 25.9 | 22.7 |
| Incidence in 2019 vs. 1997 (%) | 1004 | 592 | 1440 | 481 | 2432 | 1169 | 853 | 230 | 954 | 285 | 2043 | 564 | 905 | 396 | 1366 | 1237 | 955 | 623 | 724 | 1666 | 230 |

## Supplementary Table 2. Surgical treatment incidence per $10^5$ person-years

| year | Central Finland | Central Ostrobothnia | East Savo | Helsinki and Uusimaa | Kainuu | Kanta-Häme | Kymenlaakso | Lapland | Länsi-Pohja | North Karelia | North Savo | Northern Ostrobothnia | Pirkanmaa | Päijät-Häme | Satakunta | South Karelia | South Ostrobothnia | South Savo | Southwest Finland | Vaasa | Åland |
|---|---|---|---|---|---|---|---|---|---|---|---|---|---|---|---|---|---|---|---|---|---|
| 1997 | 12.8 | 17.9 | 2.4 | 16.4 | 15.5 | 12.8 | 10.7 | 5.9 | 16.1 | 6.9 | 9.6 | 14.8 | 16.2 | 10.1 | 12.0 | 9.7 | 9.9 | 12.8 | 14.5 | 19.4 | 4.9 |
| 1998 | 15.4 | 8.1 | 7.2 | 18.2 | 14.2 | 18.8 | 16.1 | 16.9 | 14.3 | 7.0 | 14.0 | 12.9 | 12.1 | 5.9 | 11.5 | 15.1 | 13.0 | 18.2 | 19.3 | 17.8 | 0.0 |
| 1999 | 17.4 | 11.4 | 7.2 | 20.9 | 10.0 | 13.5 | 16.1 | 20.0 | 21.6 | 21.0 | 11.6 | 19.6 | 17.3 | 14.2 | 12.0 | 15.1 | 10.6 | 17.2 | 21.6 | 19.4 | 19.4 |
| 2000 | 11.5 | 26.1 | 2.4 | 20.6 | 10.0 | 18.7 | 14.8 | 18.2 | 7.3 | 18.9 | 18.0 | 14.8 | 16.9 | 18.2 | 19.4 | 13.3 | 14.9 | 11.9 | 19.1 | 19.4 | 29.0 |
| 2001 | 12.0 | 18.0 | 9.8 | 22.9 | 15.9 | 14.2 | 16.9 | 13.2 | 18.3 | 12.6 | 17.0 | 19.2 | 16.0 | 18.8 | 22.1 | 16.0 | 16.8 | 16.3 | 22.2 | 21.7 | 24.0 |
| 2002 | 20.2 | 21.3 | 12.3 | 19.2 | 27.7 | 14.1 | 14.9 | 9.2 | 14.7 | 11.9 | 13.6 | 18.0 | 21.7 | 20.5 | 21.1 | 8.9 | 17.5 | 20.7 | 19.9 | 13.1 | 33.3 |
| 2003 | 15.4 | 21.3 | 14.8 | 19.7 | 11.7 | 20.7 | 17.6 | 18.4 | 14.8 | 12.7 | 15.6 | 14.8 | 12.4 | 24.5 | 17.4 | 15.1 | 17.5 | 15.3 | 21.4 | 17.7 | 18.9 |
| 2004 | 16.4 | 16.3 | 17.3 | 18.7 | 14.7 | 22.1 | 22.3 | 14.3 | 11.1 | 19.0 | 18.5 | 16.4 | 19.3 | 16.8 | 22.7 | 12.4 | 19.9 | 17.5 | 20.5 | 23.1 | 9.4 |
| 2005 | 12.7 | 27.7 | 32.3 | 17.9 | 5.9 | 23.4 | 13.5 | 12.2 | 12.9 | 16.8 | 17.5 | 18.6 | 17.7 | 23.2 | 29.1 | 16.0 | 13.7 | 26.3 | 20.7 | 16.1 | 32.4 |
| 2006 | 17.7 | 21.1 | 27.4 | 17.2 | 13.4 | 18.2 | 17.6 | 14.3 | 18.5 | 15.4 | 11.7 | 17.1 | 21.2 | 19.0 | 20.1 | 17.8 | 20.5 | 21.9 | 24.8 | 20.6 | 41.2 |
| 2007 | 18.1 | 16.2 | 20.1 | 17.7 | 22.4 | 27.3 | 24.4 | 19.3 | 9.2 | 18.3 | 15.1 | 22.0 | 24.1 | 16.7 | 20.1 | 16.9 | 14.9 | 8.8 | 25.2 | 11.4 | 27.2 |
| 2008 | 19.9 | 14.5 | 20.3 | 17.5 | 12.0 | 12.8 | 17.7 | 12.2 | 11.1 | 12.0 | 19.4 | 16.2 | 21.7 | 22.8 | 22.8 | 29.3 | 16.1 | 18.8 | 23.7 | 18.0 | 17.9 |
| 2009 | 11.4 | 35.4 | 22.9 | 15.4 | 25.6 | 15.5 | 19.0 | 13.2 | 27.8 | 14.8 | 14.5 | 10.8 | 15.3 | 15.3 | 24.9 | 11.6 | 17.9 | 22.2 | 14.5 | 17.9 | 26.5 |
| 2010 | 9.8 | 9.6 | 23.0 | 13.9 | 18.1 | 16.1 | 17.6 | 8.1 | 18.6 | 15.4 | 9.7 | 12.7 | 16.2 | 10.7 | 11.1 | 8.9 | 14.2 | 17.8 | 12.9 | 17.8 | 4.4 |
| 2011 | 7.8 | 16.0 | 18.0 | 12.3 | 7.6 | 11.8 | 15.6 | 7.1 | 20.5 | 11.2 | 6.8 | 9.3 | 9.9 | 8.4 | 20.2 | 8.0 | 4.3 | 12.3 | 12.3 | 13.2 | 25.8 |
| 2012 | 4.9 | 11.2 | 5.2 | 10.0 | 7.6 | 12.5 | 16.3 | 1.0 | 1.9 | 7.0 | 8.2 | 9.6 | 10.3 | 7.3 | 16.5 | 6.2 | 4.9 | 7.8 | 12.2 | 13.9 | 21.3 |
| 2013 | 6.8 | 20.8 | 13.0 | 8.9 | 6.2 | 17.3 | 12.2 | 3.0 | 5.6 | 4.2 | 6.3 | 8.9 | 13.0 | 5.6 | 13.3 | 8.0 | 6.8 | 3.4 | 11.2 | 10.2 | 0.0 |
| 2014 | 1.9 | 9.6 | 0.0 | 7.8 | 6.2 | 9.0 | 6.8 | 3.0 | 1.9 | 4.2 | 4.8 | 3.5 | 5.3 | 7.8 | 12.2 | 7.1 | 9.3 | 3.4 | 10.1 | 8.0 | 16.8 |
| 2015 | 4.8 | 9.6 | 2.7 | 7.3 | 1.6 | 6.9 | 4.8 | 1.0 | 1.9 | 7.7 | 6.7 | 4.4 | 8.3 | 3.9 | 10.7 | 7.2 | 6.2 | 5.7 | 12.3 | 6.5 | 4.2 |
| 2016 | 6.3 | 11.2 | 5.4 | 5.2 | 9.4 | 13.2 | 6.2 | 2.0 | 1.9 | 4.2 | 3.4 | 2.5 | 5.0 | 3.9 | 14.5 | 5.4 | 9.3 | 4.5 | 10.5 | 10.1 | 8.3 |
| 2017 | 2.4 | 11.2 | 2.7 | 6.0 | 3.2 | 7.0 | 3.5 | 3.0 | 1.9 | 2.1 | 2.9 | 3.7 | 4.3 | 5.6 | 7.0 | 2.7 | 7.5 | 4.6 | 3.7 | 3.6 | 8.3 |
| 2018 | 3.4 | 12.8 | 2.8 | 6.7 | 3.2 | 6.3 | 4.9 | 3.0 | 2.0 | 2.1 | 2.9 | 2.2 | 5.2 | 5.1 | 4.9 | 0.9 | 8.1 | 1.2 | 6.9 | 5.1 | 4.1 |
| 2019 | 2.9 | 9.7 | 0.0 | 6.0 | 0.0 | 4.2 | 3.5 | 1.0 | 0.0 | 2.8 | 1.0 | 4.3 | 4.9 | 4.5 | 5.5 | 6.4 | 1.9 | 3.5 | 8.4 | 3.6 | 0.0 |
| Change 2019-1997 | -9.9 | -8.3 | -2.4 | -10.4 | -15.5 | -8.6 | -7.1 | -4.9 | -16.1 | -4.1 | -8.7 | -10.5 | -11.2 | -5.6 | -6.5 | -3.4 | -8.0 | -9.3 | -6.1 | -15.8 | -4.9 |
| Incidence in 2019 vs. 1997 (%) | -78 | -46 | -100 | -63 | -100 | -67 | -67 | -83 | -100 | -59 | -90 | -71 | -69 | -55 | -54 | -35 | -81 | -73 | -42 | -81 | -100 |

## Supplement Table 3. Total incidence per $10^5$ person-years

| year | Central Finland | Central Ostrobothnia | East Savo | Helsinki and Uusimaa | Kainuu | Kanta-Häme | Kymenlaakso | Lapland | Länsi-Pohja | North Karelia | North Savo | Northern Ostrobothnia | Pirkanmaa | Päijät-Häme | Satakunta | South Karelia | South Ostrobothnia | South Savo | Southwest Finland | Vaasa | Åland |
|---|---|---|---|---|---|---|---|---|---|---|---|---|---|---|---|---|---|---|---|---|---|
| 1997 | 16.0 | 24.5 | 4.8 | 20.8 | 16.9 | 15.1 | 14.0 | 14.8 | 19.6 | 15.3 | 11.1 | 18.5 | 18.6 | 15.4 | 13.5 | 12.4 | 13.0 | 18.2 | 17.3 | 21.0 | 14.8 |
| 1998 | 20.7 | 31.0 | 7.2 | 26.8 | 21.3 | 27.1 | 21.4 | 30.8 | 19.7 | 15.3 | 24.6 | 19.8 | 15.8 | 19.5 | 14.6 | 20.4 | 18.0 | 24.7 | 26.2 | 23.2 | 14.7 |
| 1999 | 22.7 | 27.7 | 7.2 | 29.5 | 19.9 | 21.0 | 20.8 | 29.0 | 25.3 | 25.1 | 18.9 | 21.4 | 23.8 | 21.2 | 16.7 | 21.3 | 14.3 | 18.3 | 29.3 | 24.8 | 38.9 |
| 2000 | 21.5 | 39.2 | 12.2 | 30.5 | 11.5 | 26.2 | 20.2 | 20.2 | 14.5 | 23.1 | 24.8 | 26.8 | 24.1 | 27.1 | 24.6 | 22.2 | 19.9 | 17.3 | 27.8 | 24.0 | 29.0 |
| 2001 | 18.7 | 31.1 | 19.6 | 31.0 | 27.5 | 20.9 | 27.0 | 24.4 | 21.9 | 18.2 | 24.3 | 25.1 | 22.9 | 27.0 | 28.4 | 20.4 | 17.5 | 22.8 | 28.7 | 24.7 | 33.7 |
| 2002 | 35.2 | 34.3 | 27.0 | 29.0 | 40.8 | 22.3 | 18.3 | 16.4 | 22.1 | 21.8 | 22.4 | 26.7 | 30.9 | 29.8 | 24.8 | 13.3 | 23.1 | 21.7 | 25.3 | 18.5 | 38.1 |
| 2003 | 22.1 | 29.4 | 19.7 | 27.7 | 23.5 | 27.4 | 24.4 | 23.5 | 27.7 | 22.5 | 20.4 | 22.7 | 17.0 | 30.3 | 22.7 | 23.1 | 20.0 | 17.5 | 28.7 | 25.5 | 23.6 |
| 2004 | 28.1 | 31.1 | 32.2 | 28.0 | 22.1 | 30.9 | 33.9 | 23.5 | 16.6 | 29.5 | 27.7 | 21.8 | 26.9 | 24.4 | 34.3 | 18.7 | 26.2 | 24.1 | 29.1 | 30.8 | 18.7 |
| 2005 | 26.9 | 31.0 | 47.1 | 30.4 | 22.2 | 37.3 | 18.9 | 17.3 | 14.8 | 31.6 | 28.2 | 26.4 | 27.1 | 35.3 | 41.8 | 31.1 | 21.8 | 35.1 | 30.8 | 23.0 | 37.0 |
| 2006 | 29.3 | 35.8 | 32.4 | 31.8 | 37.2 | 30.5 | 29.1 | 27.5 | 22.1 | 27.4 | 20.4 | 26.2 | 30.8 | 27.7 | 32.3 | 25.7 | 29.2 | 25.2 | 31.6 | 38.8 | 45.8 |
| 2007 | 32.1 | 32.4 | 35.1 | 31.2 | 34.4 | 43.8 | 32.6 | 30.5 | 14.8 | 29.5 | 19.9 | 33.9 | 32.0 | 23.5 | 29.7 | 31.1 | 24.8 | 19.8 | 34.3 | 22.7 | 40.8 |
| 2008 | 44.3 | 29.1 | 43.0 | 35.5 | 22.5 | 33.4 | 33.3 | 25.4 | 16.7 | 21.1 | 33.0 | 31.3 | 32.9 | 38.8 | 31.3 | 45.3 | 27.9 | 26.6 | 37.8 | 29.3 | 26.9 |
| 2009 | 28.2 | 51.6 | 38.2 | 32.1 | 39.1 | 33.1 | 31.2 | 25.3 | 35.3 | 19.7 | 26.7 | 30.1 | 29.5 | 30.7 | 36.6 | 22.2 | 35.3 | 34.4 | 32.1 | 26.8 | 44.2 |
| 2010 | 26.1 | 30.5 | 51.2 | 29.5 | 34.8 | 32.9 | 30.5 | 24.3 | 29.7 | 27.4 | 17.9 | 33.1 | 29.2 | 28.3 | 23.3 | 22.2 | 22.2 | 28.9 | 30.9 | 31.1 | 17.5 |
| 2011 | 30.4 | 27.2 | 46.3 | 35.1 | 28.9 | 30.6 | 25.1 | 27.2 | 37.3 | 39.3 | 31.4 | 31.2 | 29.3 | 29.8 | 43.5 | 31.2 | 24.7 | 27.9 | 34.6 | 26.5 | 43.1 |
| 2012 | 21.9 | 28.8 | 23.3 | 38.8 | 38.2 | 38.1 | 36.0 | 47.4 | 44.9 | 40.7 | 34.3 | 43.8 | 31.9 | 27.5 | 34.0 | 47.2 | 30.8 | 38.1 | 30.3 | 37.3 | 51.2 |
| 2013 | 38.8 | 59.1 | 44.3 | 39.3 | 30.8 | 40.1 | 35.4 | 34.3 | 39.5 | 40.7 | 36.1 | 40.0 | 37.9 | 27.4 | 44.6 | 44.5 | 38.8 | 28.1 | 36.5 | 42.2 | 33.9 |
| 2014 | 34.4 | 54.3 | 28.9 | 34.9 | 34.1 | 33.9 | 36.2 | 40.3 | 30.3 | 41.4 | 33.2 | 31.9 | 34.7 | 34.2 | 40.5 | 32.1 | 38.3 | 34.9 | 41.1 | 29.7 | 71.5 |
| 2015 | 39.2 | 33.5 | 37.1 | 32.7 | 26.6 | 39.4 | 38.4 | 39.3 | 32.5 | 32.3 | 30.8 | 30.8 | 35.7 | 32.5 | 33.6 | 31.3 | 32.2 | 39.6 | 36.2 | 28.1 | 16.7 |
| 2016 | 52.0 | 33.5 | 53.5 | 32.4 | 34.6 | 45.0 | 42.7 | 27.3 | 26.9 | 30.3 | 26.9 | 32.5 | 33.4 | 36.4 | 35.9 | 26.1 | 31.7 | 36.4 | 40.5 | 36.0 | 41.6 |
| 2017 | 36.5 | 52.8 | 40.6 | 32.7 | 34.9 | 29.2 | 24.9 | 30.3 | 40.7 | 34.6 | 28.9 | 29.9 | 31.4 | 34.2 | 36.6 | 37.9 | 37.4 | 44.7 | 31.6 | 36.8 | 37.1 |
| 2018 | 28.8 | 53.0 | 30.4 | 33.5 | 33.7 | 44.0 | 35.0 | 33.4 | 31.3 | 36.2 | 33.3 | 29.1 | 30.9 | 44.4 | 32.5 | 29.0 | 50.1 | 27.7 | 30.5 | 41.9 | 53.2 |
| 2019 | 38.3 | 54.8 | 36.7 | 31.1 | 35.6 | 32.9 | 35.3 | 30.4 | 37.6 | 34.9 | 32.0 | 28.3 | 28.9 | 31.0 | 28.3 | 41.9 | 34.5 | 42.1 | 31.3 | 31.0 | 32.6 |
| Change 2019-1997 | 22.2 | 30.3 | 31.9 | 10.4 | 18.7 | 17.8 | 21.3 | 15.5 | 18.0 | 19.6 | 20.9 | 9.9 | 10.4 | 15.6 | 14.8 | 29.5 | 21.5 | 23.9 | 14.1 | 10.1 | 17.8 |
| Incidence in 2019 vs. 1997 (%) | 139 | 124 | 670 | 50 | 111 | 118 | 152 | 105 | 92 | 129 | 188 | 54 | 56 | 101 | 109 | 238 | 166 | 132 | 82 | 48 | 120 |

# Supplement Table 4. Non-surgical proportion

| year | Central Finland | Central Ostrobothnia | East Savo | Helsinki and Uusimaa | Kainuu | Kanta-Häme | Kymenlaakso | Lapland | Länsi-Pohja | North Karelia | North Savo | Northern Ostrobothnia | Pirkanmaa | Päijät-Häme | Satakunta | South Karelia | South Ostrobothnia | South Savo | Southwest Finland | Vaasa | Åland |
|---|---|---|---|---|---|---|---|---|---|---|---|---|---|---|---|---|---|---|---|---|---|
| 1997 | 20 | 27 | 50 | 21 | 8 | 15 | 24 | 60 | 18 | 55 | 13 | 20 | 13 | 35 | 12 | 21 | 24 | 29 | 16 | 7 | 67 |
| 1998 | 26 | 74 | 0 | 32 | 33 | 31 | 25 | 45 | 27 | 55 | 43 | 35 | 23 | 70 | 21 | 26 | 28 | 26 | 26 | 23 | 100 |
| 1999 | 23 | 59 | 0 | 29 | 50 | 36 | 23 | 31 | 14 | 17 | 38 | 8 | 27 | 33 | 28 | 29 | 26 | 6 | 26 | 22 | 50 |
| 2000 | 46 | 33 | 80 | 32 | 13 | 29 | 27 | 10 | 50 | 18 | 27 | 45 | 30 | 33 | 21 | 40 | 25 | 31 | 31 | 19 | 0 |
| 2001 | 36 | 42 | 50 | 26 | 42 | 32 | 38 | 46 | 17 | 31 | 30 | 24 | 30 | 30 | 22 | 22 | 4 | 29 | 23 | 13 | 29 |
| 2002 | 43 | 38 | 55 | 34 | 32 | 37 | 19 | 44 | 33 | 45 | 39 | 32 | 30 | 31 | 15 | 33 | 24 | 5 | 21 | 29 | 13 |
| 2003 | 30 | 28 | 25 | 29 | 50 | 24 | 28 | 22 | 47 | 44 | 24 | 35 | 27 | 19 | 23 | 35 | 13 | 13 | 25 | 30 | 20 |
| 2004 | 42 | 47 | 46 | 33 | 33 | 29 | 34 | 39 | 33 | 36 | 33 | 25 | 28 | 31 | 34 | 33 | 24 | 27 | 29 | 25 | 50 |
| 2005 | 53 | 11 | 32 | 41 | 73 | 37 | 29 | 29 | 13 | 47 | 38 | 29 | 35 | 34 | 30 | 49 | 37 | 25 | 33 | 30 | 13 |
| 2006 | 40 | 41 | 15 | 46 | 64 | 40 | 40 | 48 | 17 | 44 | 43 | 35 | 31 | 31 | 38 | 31 | 30 | 13 | 22 | 47 | 10 |
| 2007 | 44 | 50 | 43 | 43 | 35 | 38 | 25 | 37 | 38 | 38 | 24 | 35 | 24 | 29 | 32 | 46 | 40 | 56 | 27 | 50 | 33 |
| 2008 | 55 | 50 | 53 | 51 | 47 | 62 | 47 | 52 | 33 | 43 | 41 | 48 | 34 | 41 | 27 | 35 | 42 | 29 | 37 | 38 | 33 |
| 2009 | 60 | 31 | 40 | 52 | 35 | 53 | 39 | 48 | 21 | 25 | 45 | 64 | 48 | 50 | 32 | 48 | 49 | 35 | 55 | 33 | 40 |
| 2010 | 62 | 68 | 55 | 53 | 48 | 51 | 42 | 67 | 38 | 44 | 46 | 62 | 45 | 62 | 52 | 60 | 36 | 38 | 58 | 43 | 75 |
| 2011 | 74 | 41 | 61 | 65 | 74 | 61 | 38 | 74 | 45 | 71 | 78 | 70 | 66 | 72 | 54 | 74 | 83 | 56 | 64 | 50 | 40 |
| 2012 | 78 | 61 | 78 | 74 | 80 | 67 | 55 | 98 | 96 | 83 | 76 | 78 | 68 | 73 | 52 | 87 | 84 | 79 | 60 | 63 | 58 |
| 2013 | 83 | 65 | 71 | 77 | 80 | 57 | 65 | 91 | 86 | 90 | 83 | 78 | 66 | 80 | 70 | 82 | 83 | 88 | 69 | 76 | 100 |
| 2014 | 94 | 82 | 100 | 78 | 82 | 73 | 81 | 93 | 94 | 90 | 86 | 89 | 85 | 77 | 70 | 78 | 76 | 90 | 75 | 73 | 76 |
| 2015 | 88 | 71 | 93 | 78 | 94 | 82 | 88 | 97 | 94 | 76 | 78 | 86 | 77 | 88 | 68 | 77 | 81 | 86 | 66 | 77 | 75 |
| 2016 | 88 | 67 | 90 | 84 | 73 | 71 | 85 | 93 | 93 | 86 | 88 | 92 | 85 | 89 | 60 | 79 | 71 | 88 | 74 | 72 | 80 |
| 2017 | 93 | 79 | 93 | 82 | 91 | 76 | 86 | 90 | 95 | 94 | 90 | 88 | 86 | 84 | 81 | 93 | 80 | 90 | 88 | 90 | 78 |
| 2018 | 88 | 76 | 91 | 80 | 90 | 86 | 86 | 91 | 94 | 94 | 91 | 93 | 83 | 89 | 85 | 97 | 84 | 96 | 77 | 88 | 92 |
| 2019 | 93 | 82 | 100 | 81 | 100 | 87 | 90 | 97 | 100 | 92 | 97 | 85 | 83 | 85 | 81 | 85 | 95 | 92 | 73 | 88 | 100 |
| Change 2019-1997 (%-points) | 73 | 56 | 50 | 60 | 92 | 72 | 66 | 37 | 82 | 37 | 84 | 65 | 70 | 51 | 69 | 63 | 71 | 62 | 57 | 81 | 33 |
| Proportion in 2019 vs. 1997 (%) | 363 | 209 | 100 | 288 | 1100 | 482 | 278 | 61 | 450 | 68 | 643 | 332 | 545 | 147 | 600 | 296 | 297 | 212 | 354 | 1093 | 50 |
